# Women's empowerment and its association with intimate partner violence in sub-Saharan Africa: a study using recent demographic and health surveys

**DOI:** 10.3389/fgwh.2026.1929589

**Published:** 2026-10-07

**Authors:** John Mugisha, Theophile Dushimirimana, Munawar Harun Koray

**Affiliations:** 1 Ministry of Education, Kigali, Rwanda; 2 Southern Medical University, Guangzhou, China; 3 Rwanda Implementation Science and Health Systems Labs, Kigali, Rwanda; 4 Upper West Regional Health Directorate, Ghana Health Service, Wa, Ghana

**Keywords:** health equality, intimate partner violence, sub-Saharan Africa, SWPER index, women’s empowerment

## Abstract

**Background:**

Intimate partner violence (IPV) remains a major public health and human rights concern in sub-Saharan Africa (SSA). This study examined the association between multidimensional empowerment and IPV among married or cohabiting women using the Survey-based Women's Empowerment (SWPER) Global Index.

**Methodology:**

We conducted a cross-sectional analysis of the most recent Demographic and Health Survey data collected between 2010 and 2023 in 21 Sub-Saharan African countries. The study included 90,807 married or cohabiting women aged 15–49 years. Women's empowerment was measured using the three SWPER Global domains. Survey-weighted bivariate analyses and multivariable logistic regression were used to examine associations with IPV, with adjusted odds ratios and 95% confidence intervals reported.

**Results:**

Pooled IPV prevalence was 12.4% (2.0% in Mauritania to 30.8% in Sierra Leone). High empowerment in the attitudes-towards-violence domain (aOR = 0.59; 95% CI: 0.55–0.63) and social-independence domain (aOR = 0.84; 95% CI: 0.77–0.92) was associated with lower odds of IPV. Decision-making empowerment was not independently associated with IPV (high vs. low: aOR = 1.05; 95% CI: 0.98–1.13). Primary education was associated with slightly higher odds of IPV (aOR = 1.08; 95% CI: 1.00–1.16), and higher education was associated with lower odds (aOR = 0.75; 95% CI: 0.61–0.93). Older age (aORs = 1.37–1.96), cohabitation (aOR = 1.11; 95% CI: 1.04–1.19), primary education (aOR = 1.08; 95% CI: 1.00–1.16), parity of two or more (aORs = 1.39–1.52), several occupational groups (aORs = 1.17–1.45), and partner's secondary education (aOR = 1.23; 95% CI: 1.13–1.34) were associated with higher odds of IPV. In contrast, Islamic affiliation (aOR = 0.88; 95% CI: 0.82–0.96), richer or richest wealth status (aORs = 0.91 and 0.81) were associated with lower odds.

**Conclusion:**

Women's empowerment is associated with IPV in a multidimensional manner. Prevention efforts should support women's agency while also addressing unequal power within relationships, norms that tolerate violence, and barriers to survivor-centered services.

## Introduction

Intimate partner violence (IPV) is a major public health problem and one of the most prevalent forms of women's rights violations in the world. IPV is defined as physical, sexual, or psychological abuse perpetrated by a current or former intimate partner within an intimate relationship (1, 2). Despite growing attention to gender equality, IPV remains widespread and has serious consequences for women's physical, reproductive, and mental health, family well-being, and participation in society (3).

The marked variation in IPV across countries and types of violence in sub-Saharan Africa shows that IPV is more than just a private family issue. It is shaped by the broader conditions in which relationships occur, including gender norms, economic pressures, unequal power within relationships, legal protections, and community attitudes that either tolerate or challenge violence against women (4–8).

Women who experience IPV may face both immediate and long-term health consequences, including injuries, chronic pain, unintended pregnancies, sexually transmitted infections, depression, anxiety, and other adverse physical and mental health outcomes (9, 10). These effects extend beyond individual women to their families, communities, and future generations. IPV may limit women's mobility, reduce their ability to participate fully in social and economic life, weaken economic security, and disrupt family well-being (11, 12). For this reason, reducing IPV is closely aligned with the Sustainable Development Goals, particularly SDG 3 on optimal health and well-being and SDG 5 on gender equality, including Target 5.2, which aims to eliminate all forms of violence against women and girls (1, 13).

Women's empowerment is often viewed as an important pathway to reducing IPV, but the relationship is not always straightforward. Greater empowerment may help women recognize abuse, participate in household decisions, access resources, seek support, and resist coercive control. However, evidence from low- and middle-income countries suggests that some forms of empowerment can have mixed associations with IPV. This is especially when women's increasing autonomy challenges established gender hierarchies without similar changes in male attitudes, household power relations, and community norms (14, 15). In patriarchal settings, traditional household authority may perceive women's education, autonomy, employment, or social mobility as a threat (5, 14).

Globally, an estimated 25.8% of women of reproductive age have experienced physical or sexual IPV during their lifetime, although prevalence varies substantially across sub-Saharan African settings (4, 16).

A limitation of much of the earlier literature is that a single indicator, such as education, employment, household wealth, or decision-making, has often measured women's empowerment. Although these indicators are useful, they may not fully capture empowerment as a multidimensional process (17). The survey-based Women's Empowerment Global Index offers a more comprehensive approach by measuring empowerment across three complementary domains: attitudes towards violence, social independence, and decision-making (18, 19). These domains make it possible to distinguish women's rejection of violence-supportive norms, their broader social position, and their participation in household decisions.

The use of SWPER is particularly appropriate for the present study because the index was developed and validated using DHS data and later expanded for use across low- and middle-income countries (18, 19). In sub-Saharan Africa, its three domains are well-suited to examining how empowerment operates across different levels of social life. Attitudes towards violence capture women's acceptance or rejection of wife-beating and reflect internalized gender norms. Social independence captures women's broader social positioning, including education, access to information, and timing of marriage and childbearing. Decision-making reflects women's participation in household choices and, indirectly, the negotiation of power within intimate relationships.

This study used recent Demographic and Health Survey data from 21 sub-Saharan African countries to examine the association between the three SWPER domains, attitudes towards violence, social independence, and decision-making, and IPV. The analysis adjusted for selected sociodemographic, household, partner, and contextual characteristics (5, 20). By examining empowerment as a multidimensional construct, the study sought to clarify whether its different domains relate to IPV in similar or different ways. Understanding these relationships may help inform empowerment and IPV-prevention strategies that are responsive to the diverse contexts represented in the study.

## Study methods

### Study design and data source

This cross-sectional study pooled the most recent eligible Demographic and Health Survey conducted in each of 21 sub-Saharan African countries between 2010 and 2023. The pooled analysis was intended to estimate average associations across the included surveys using standardized DHS measures, rather than to represent all countries at a single point in time. Because each country contributed only one survey, the survey year was closely tied to the country and was not entered separately in the country-adjusted sensitivity model. The DHS is a nationally representative survey conducted in more than 90 low- and middle-income countries worldwide over a 5-year period (21, 22). The DHS program employs a multistage sampling method to select approximately 5,000 to 32,000 participants. The sample is generally representative at the national, regional, and residence (urban-rural) levels.

The DHS data are collected from participants using a standardized, structured questionnaire that covers various health indicators. These include maternal demographics, household and community characteristics, child-related factors, and malnutrition. A comprehensive explanation of the sampling methods can be found on the DHS website: https://www.dhsprogram.com/Methodology/Survey-Types/DHS-Methodology.cfm (21) and in the relevant literature (23). The pooled survey files initially included 413,345 women aged 15–49 years. The analytic sample was restricted to women who were married or cohabiting, were selected for and completed the domestic violence module, and had sufficient information to derive all three SWPER domains and the variables included in the regression analysis (19). The analysis used a final pooled dataset of 90,807 married or cohabiting women who were selected for and completed the DHS domestic violence module and had sufficient information to derive the IPV outcome and the three SWPER domains. The final analytic file retained the women who met these predefined eligibility criteria (Figure 1). Complete-case analysis was used for the regression models. Because religion was unavailable for some surveys, the exact number of women and countries included in the primary model differed from the country-adjusted sensitivity analysis. Figure 1 presents the final descriptive and model-specific analytic samples.

**Figure 1:**
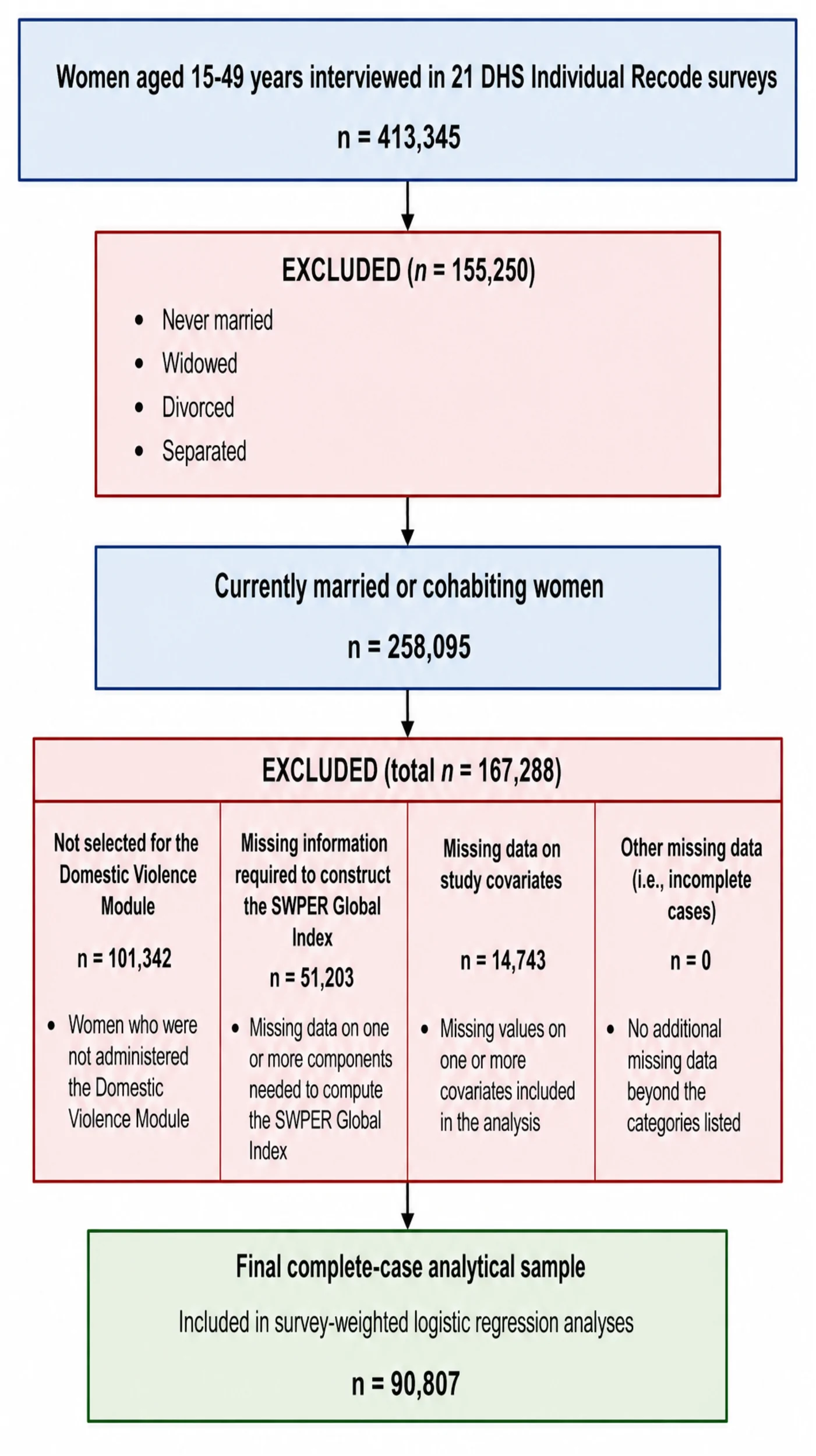
Flow chart showing the sample selection.

### Study variables

#### The outcome variable

The outcome was intimate partner violence during the 12 months preceding the survey. A binary composite variable was constructed from women's reported experience of physical, sexual, or emotional violence by any intimate partner during this period. Women who reported at least one of the three forms of violence were coded 1 (experienced IPV), while women who reported none were coded 0 (did not experience IPV). The analysis was restricted to married or cohabiting women who were selected for and completed the DHS domestic violence module. Before pooling, the component variables were reviewed across the included surveys to confirm the comparability of their definitions, response categories, and 12-month reference periods and were harmonized using the corresponding DHS recode items. The physical, sexual, and emotional IPV outcomes presented separately in Tables 2, 3 were also defined using the same 12-month reference period. The composite outcome was coded as 1 when a woman reported at least one of these three forms of IPV during the previous 12 months and as 0 when she reported none.

#### Primary exposure

Empowerment of women was measured using the SWPER Global Index (19), a validated multidimensional instrument developed from DHS data, which comprises three key domains: (i) attitudes towards violence, (ii) social independence, and (iii) decision-making. The “Attitudes towards violence” domain evaluates a woman's view on whether a husband's physical violence against his wife is justifiable in certain situations. The “social independence” domain examines a woman's access to information, educational attainment, age at marriage and childbirth, and differences in age and education relative to her partner. The “Decision-making” domain assesses who holds decision-making power in the household, including decisions related to the woman's work. Each domain was classified into low, medium, and high empowerment using established cutoffs (18) derived from 14 questions in the DHS women's questionnaire. Ewerling and colleagues provide a detailed description of the design and methodology of this index (18, 19). The STATA do.file for calculating the SWPER index is available at https://goo.gl/isGonn (18).

#### Other independent variables (covariates)

The other independent variables include socio-demographic characteristics of the participants obtained from the literature such as age, marital status, religion, education, occupation, parity, wealth index, place of residence, partner's age, and partner's education (24–26). The variables are summarized in Supplementary Table S2.

### Data analysis

The analysis included descriptive statistics, survey-adjusted bivariate tests, and multivariable logistic regression. In the descriptive analysis, we assessed the prevalence of IPV, women's empowerment, and the distribution of covariates across all 21 SSA countries included in the study. The distribution of women's empowerment levels was also assessed across all three domains.

In the second stage, survey-adjusted Pearson chi-square tests were used to examine associations between the three SWPER domains, selected covariates, and each past-year IPV outcome. Physical, sexual, and emotional IPV were examined separately, together with the composite outcome representing experience of at least one of these forms during the previous 12 months. The level of significance in the bivariate analysis was set at *p* ≤ 0.05. The primary analysis used survey-weighted multivariable logistic regression to estimate pooled associations between the SWPER domains and the composite past-year IPV outcome. A preliminary two-level random-intercept logistic model (women nested within countries) was fitted to assess clustering by country; this yielded an intraclass correlation coefficient of 18.7% and a likelihood ratio test comparing the multilevel and single-level models was not statistically significant (*χ*^2^ = 2517.9, df = 1, *p* < 0.0001), indicating that clustering by country accounted for a negligible share of the variance in IPV. Standard survey-weighted logistic regression, with country entered as a fixed covariate in the sensitivity model, was therefore considered an appropriate and more parsimonious approach for the pooled analysis.

### Model diagnostic and fitness

The Hosmer-Lemeshow goodness-of-fit test indicated that the logistic regression model fit the data adequately (*χ*^2^ = 13.85, df = 8, *p* = 0.086), suggesting no evidence of poor model fit. Model discrimination was assessed using the area under the receiver operating characteristic curve (AUC = 0.624) and McFadden's pseudo *R*^2^ was 0.04, with 41.7% of observations correctly classified. Complete-case analysis was used for all regression models; cases with missing values on any covariate, most commonly religion, which was not collected in a small number of surveys, were excluded from the relevant model. Of the 90,807 women in the descriptive sample, (98%) had complete data for the primary regression model.

The variance inflation factor (VIF) was used to assess multicollinearity among the study variables. There was no multicollinearity among the SWPER domains (min = 1.04, max = 1.05 and mean VIF = 1.04). There was no evidence of overall multicollinearity in the adjusted regression model (min = 1.0, max = 2.9, and mean = 1.6). The study analysis accounted for complex DHS survey designs using sample weights (v005), primary sampling units (v021), and strata (v023) using STATA version 18. The analysis was performed, considering the complex survey design implemented by the DHS program. The STATA command “svy” was used in addition to the typical commands to account for the weighted data. The bar graph was designed using ggplot in RStudio; the cluster graphs were designed using Microsoft Excel; and ArcMap version 10.2 was used to design the symbology map.

### Ethical consideration

This study used publicly available secondary data from the Demographic and Health Surveys (DHS) Program. Ethical approval for each DHS survey was obtained by the respective national ethics committees and the ICF Institutional Review Board. Permission to use the datasets was granted by the DHS Program. Because this study involved secondary analysis of anonymized public data, additional ethical approval and informed consent were not required. The data are available free of charge to registered users through the DHS Program website (https://dhsprogram.com/Data/).

## Results

The study included 90,807 women. Table 1 presents their sociodemographic characteristics. The table shows a predominantly young population (59.8% aged 20–34) and a predominantly rural composition (62.4%). The vast majority are married (75.8%) and have low levels of formal education, with 66.6% having no formal education or only a primary education. The cohort is primarily employed in agriculture (28.7%) or sales (17.1%), with 29.5% not working. High fertility was observed, with 42.1% of women having a parity of 4 or more children. The wealth index is relatively evenly distributed across quintiles (approx. 20% each).

**Table 1:** Sociodemographic characteristics of participants (*n* = 90,807).

Demographic characteristics	Frequency (*n* = 90,807)	Percent (%)
Age		
15–19	4,933	5.4
20–24	15,797	17.4
25–29	20,322	22.4
30–34	18,132	20.0
35–39	14,583	16.1
40–44	9,907	10.9
45–49	7,143	7.9
Marital status		
Married	68,857	75.8
Cohabitation	21,960	24.2
Religion		
Christianity	56,294	62.0
Islam	20,631	22.7
No religion	2,431	2.7
Other religion	1,924	2.1
Traditional/Voodoo	1,490	1.6
Missing	8,047	8.9
Education		
No formal education	31,115	34.3
Primary	29,332	32.3
Secondary	24,796	27.3
Higher	5,574	6.1
Occupation		
Not work	26,744	29.5
Managerial	5,452	6.0
Clerical	1,060	1.2
Sales	15,497	17.1
Agriculture	26,078	28.7
Other	1,732	1.9
Household and domestic	7,328	8.1
Manual works	6,926	7.6
Parity		
0	5,400	5.9
1	14,308	15.8
2	17,175	18.9
3	15,708	17.3
4+	38,226	42.1
Wealth index		
Poorest	18,030	19.9
Poorer	18,013	19.8
Middle	18,088	19.9
Richer	18,762	20.7
Richest	17,924	19.7
Place of residence		
Urban	34,170	37.6
Rural	56,647	62.4
Partner's age		
Less than 21	989	1.1
21–39	51,162	56.3
40–59	34,582	38.1
60 and above	4,085	4.5
Partner's education		
No formal education	27,363	30.1
Primary	26,708	29.4
Secondary	27,914	30.7
Higher	8,711	9.6
Don't know	121	0.1

### Prevalence of IPV

The overall IPV prevalence across the 21 countries is approximately 12.4%. The highest IPV prevalence was observed in Sierra Leone, where approximately 30.8% of women reported experiencing it. This was followed by Liberia, with 26.3% of women affected. Mauritania had the lowest IPV prevalence at 2.0%. Other countries with relatively low IPV prevalence include South Africa (7.6%), Malawi (7.8%), and Burundi (7.5%). This data is presented in Figure 2.

**Figure 2:**
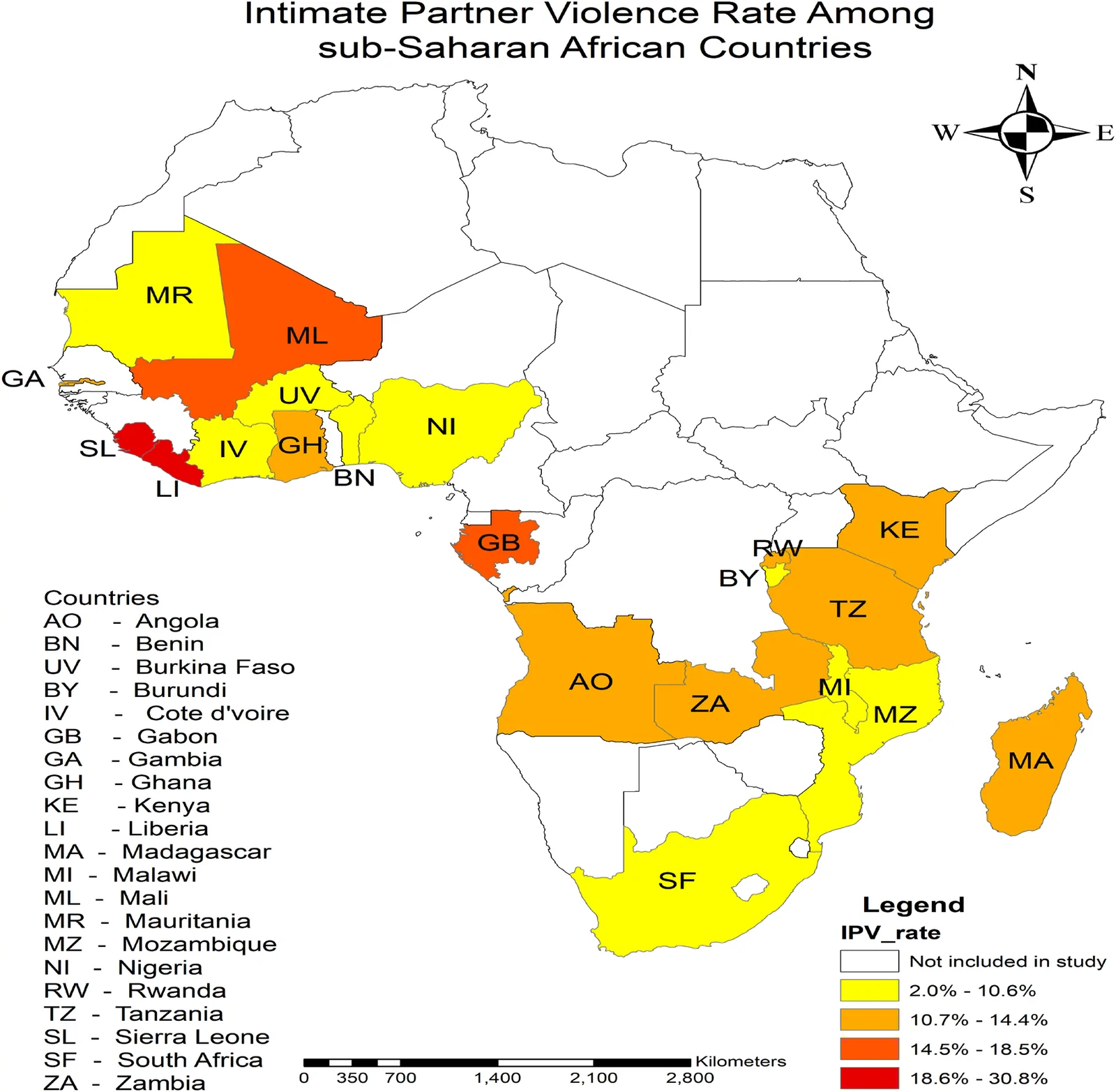
Map of Africa showing rates of IPV (any physical, sexual, or emotional IPV in the past 12 months).

### Women's empowerment levels

Empowerment levels differed across countries in all three SWPER domains (*p* < 0.001). High empowerment in attitudes towards violence was most common in South Africa (95.2%), Malawi (84.5%), and Ghana (79.9%), and least common in Mali (18.9%). High decision-making empowerment was most common in South Africa (77.9%), Rwanda (55.3%), and Gabon (53.2%), while high social independence was most common in South Africa (86.9%), Madagascar (76.3%), and Kenya (64.7%). The country-level distribution of low, medium, and high empowerment across the three SWPER domains is shown in Figure 3.

**Figure 3:**
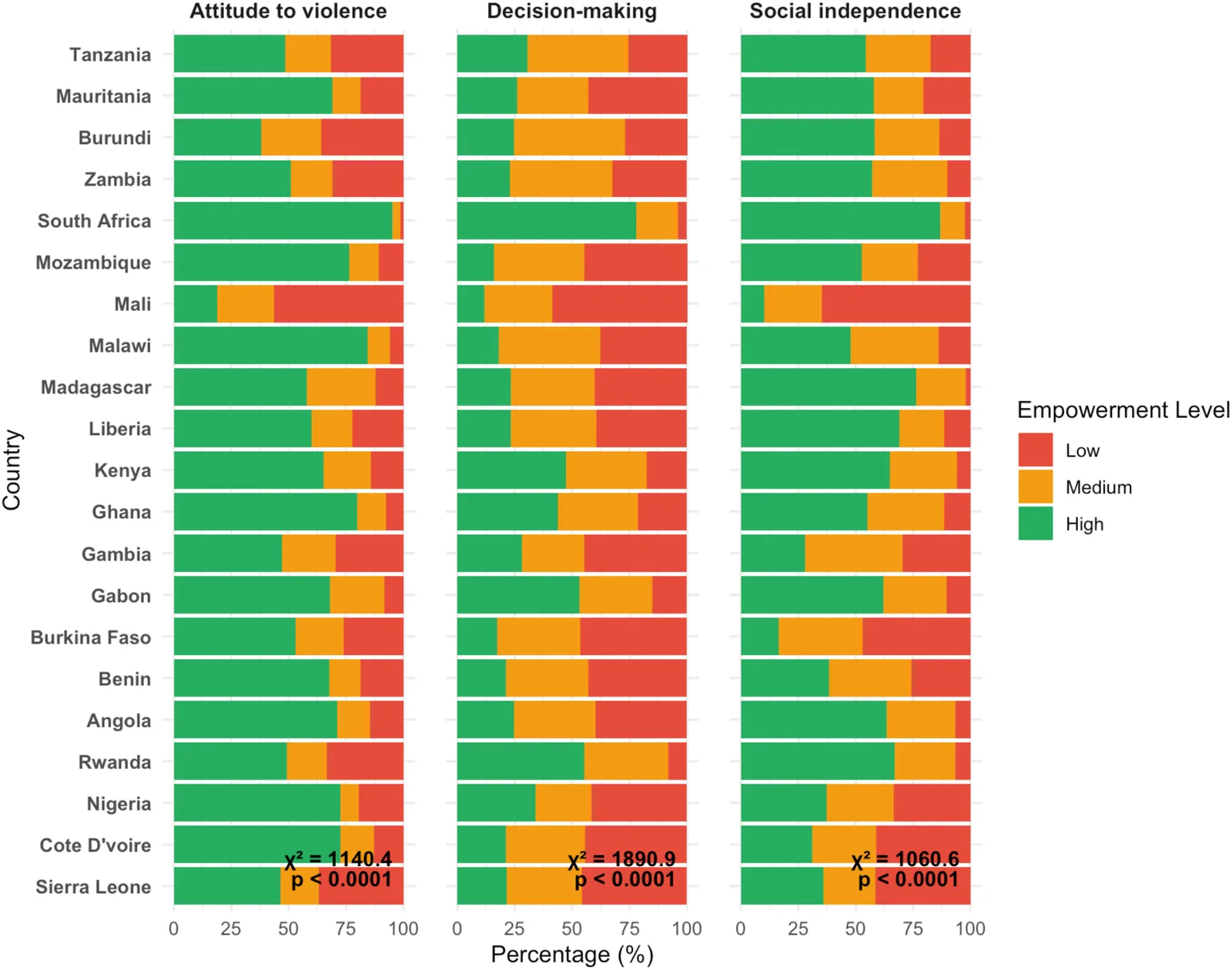
Distribution of women's empowerment levels across 21 sub-Saharan African countries by SWPER Global domain.

Table 2 presents the associations between the three SWPER domains and physical, sexual, emotional, and any IPV during the 12 months preceding the survey: (*χ*^2^ values: 184.8–606.; *p* < 0.001). Regarding physical violence, 22.7% of women with high empowerment experience violence, compared to 38.3% for low empowerment. Also, 8.2% of women with high empowerment in the attitude toward violence domain experienced sexual violence, compared to 15.2% of those with low empowerment. Emotional violence was predominantly in the attitude toward violence domain, with women with high empowerment (25.5%) experiencing it compared to 36.0% for low empowerment.

**Table 2:** Associations between women's empowerment and past-year IPV outcomes**.**

Women's empowerment	Physical IPV in the past 12 months		Sexual IPV in the past 12 months		Emotional IPV in the past 12 months		Any physical, sexual, or emotional IPV in the past 12 months	
	No	Yes	No	Yes	No	Yes	No	Yes
	*χ*^2^ value	*p*-value	*χ*^2^ value	*p*-value	*χ*^2^ value	*p*-value	*χ*^2^ value	*p*-value
Attitude to violence	**606.5**	**<0.0001**	**266.5**	**<0.0001**	**274.8**	**<0.0001**	**184.8**	**<0.0001**
Lower empowerment	11,910 (61.7)	7,402 (38.3)	16,375 (84.8)	2,937 (15.2)	12,351 (64.0)	6,961 (36.0)	16,143 (83.6)	3,169 (16.4)
Medium empowerment	10,230 (64.5)	5,639 (35.5)	13,642 (86.0)	2,227 (14.0)	10,383 (65.4)	5,486 (34.6)	13,418 (84.6)	2,451 (15.4)
High empowerment	43,027 (77.3)	12,609 (22.7)	51,060 (91.8)	4,576 (8.2)	41,435 (74.5)	14,201 (25.5)	49,900 (89.7)	5,735 (10.3)
Social independence	**116.2**	**<0.0001**	**216.0**	**<0.0001**	**197.6**	**<0.0001**	**82.8**	**<0.0001**
Lower empowerment	22,244 (70.5)	9,316 (29.5)	28,129 (89.1)	3,431 (10.9)	21,841 (69.2)	9,718 (30.8)	27,414 (86.9)	4,145 (13.1)
Medium empowerment	22,560 (69.2)	10,058 (30.8)	28,596 (87.7)	4,022 (12.3)	22,627 (69.4)	9,991 (30.6)	28,326 (86.8)	4,292 (13.2)
High empowerment	20,363 (76.4)	6,277 (23.6)	24,353 (91.4)	2,287 (8.6)	19,701 (74.0)	6,939 (26.0)	23,721 (89.0)	2,918 (11.0)
Decision making	**147.7**	**<0.0001**	**252.2**	**<0.0001**	**224**.**1**	**<0.0001**	**20.8**	**<0.01**
Lower empowerment	14,138 (73.8)	5,013 (26.2)	17,361 (90.7)	1,790 (9.3)	13,337 (69.6)	5,814 (30.4)	16,710 (87.2)	2,442 (12.8)
Medium empowerment	18,408 (69.1)	8,248 (30.9)	23,128 (86.8)	3,529 (13.2)	18,049 (67.7)	8,607 (32.3)	23,152 (86.9)	3,504 (13.1)
High empowerment	32,620 (72.5)	12,389 (27.5)	40,588 (90.2)	4,421 (9.8)	32,783 (72.8)	12,227 (27.2)	39,600 (88.0)	5,410 (12.0)

Note: Bold values indicate the *χ*² statistics and corresponding *p*-values.

Social independence was significantly associated with all forms of violence (*χ*^2^ values: 82.8–216.0, *p* < 0.001). Specifically, most of the women experienced emotional violence, with 26.0% of women with high empowerment experiencing it, compared to 30.8% of those with low empowerment. Sexual violence was the least experienced type of violence, with 8.6% of women with high empowerment experiencing violence, compared to 10.9% for low empowerment.

In the bivariate analysis, decision-making empowerment was associated with the different forms of IPV. However, the pattern was inconsistent: the proportion reporting IPV did not progressively decrease as decision-making empowerment increased. After adjustment for the other SWPER domains and covariates, neither medium nor high decision-making empowerment was independently associated with IPV. *χ*^2^ values: 20.8–252.2, *p* < 0.001 for physical, sexual, and emotional violence, and *p* < 0.01 for “Any physical, sexual, or emotional IPV in the past 12 months.” Physical violence was common among 27.5% of women with high empowerment in decision-making, compared to 26.2% for those with low empowerment. Approximately 9.8% of women with high empowerment experienced sexual violence, compared to 9.3% for low empowerment, as shown in Table 2.

Table 3 presents the associations between the selected covariates and the three forms of past-year IPV, together with the composite measure of any past-year IPV. Several of the covariates show highly significant associations with all forms of violence against women (physical, sexual, emotional, and any violence) (*p* < 0.05). Age strongly associates with violence prevalence (*χ*^2^ = 43.0–291.6, *p* < 0.001). Physical and emotional violence rise progressively with age, peaking sharply in the 40–44 age group (30.1% physical, 31.3% emotional) before slightly declining. Marital status is significantly linked to all violence types (*χ*^2^ = 10.2–187.4, *p* ≤ 0.041). Cohabiting women face substantially higher risks than married women, reporting 31.9% physical violence (vs. 27.1% married). Religion exhibits one of the strongest associations (*χ*^2^ up to 816.7, *p* < 0.001). Christian women report the highest physical violence (32.0%). Education among both women and their partners was significantly associated with intimate partner violence (*χ*^2^ = 159.7–754.0, *p* < 0.001). Women with higher education experience 16.0% physical and 5.0% sexual violence, compared to 32.8% and 13.7% among those with primary education. Violence was significantly associated with wealth index (*p* < 0.001), with 30.1% in the poorest quintile vs. 22.4% in the richest. Rural women experience higher rates of physical violence (29.5%) compared to urban women (26.1%), as well as higher rates of sexual violence (11.9% vs. 8.8%). Partner's age was significantly associated with violence (*χ*^2^ = 33.6–113.0, *p* < 0.001), with emotional violence peaking at 31.0% when partners are aged 40–59. This is presented in Table 3.

**Table 3:** Associations between selected covariates and past-year IPV outcomes.

Covariates	Physical IPV in the past 12 months		Sexual IPV in the past 12 months		Emotional IPV in the past 12 months		Any physical, sexual, or emotional IPV in the past 12 months	
	No	Yes	No	Yes	No	Yes	No	Yes
	*χ*^2^ value	*p*-value	*χ*^2^ value	*p*-value	*χ*^2^ value	*p*-value	*χ*^2^ value	*p*-value
Age	**201.2**	**<0.0001**	**43.0**	**<0.001**	**291.6**	**<0.0001**	**215.1**	**<0.0001**
15–19	3,909 (79.2)	1,025 (20.8)	4,492 (91.0)	442 (9.0)	3,916 (79.4)	1,018 (20.6)	4,558 (92.4)	375 (7.6)
20–24	11,616 (73.5)	4,181 (26.5)	14,037 (88.9)	1,761 (11.1)	11,582 (73.3)	4,215 (26.7)	14,089 (89.2)	1,708 (10.8)
25–29	14,498 (71.3)	5,825 (28.7)	18,145 (89.3)	2,178 (10.7)	14,246 (70.1)	6,077 (29.9)	17,771 (87.4)	2,551 (12.6)
30–34	12,825 (70.7)	5,307 (29.3)	16,099 (88.8)	2,033 (11.2)	12,547 (69.2)	5,585 (30.8)	15,751 (86.9)	2,381 (13.1)
35–39	10,267 (70.4)	4,316 (29.6)	12,933 (88.7)	1,650 (11.3)	10,107 (69.3)	4,476 (30.7)	12,667 (86.9)	1,916 (13.1)
40–44	6,927 (69.9)	2,980 (30.1)	8,927 (90.1)	979 (9.9)	6,811 (68.7)	3,096 (31.3)	8,440 (85.2)	1,466 (14.8)
45–49	5,125 (71.8)	2,017 (28.2)	6,444 (90.2)	698 (9.8)	4,960 (69.4)	2,182 (30.6)	6,184 (86.6)	958 (13.4)
Marital status	**187.4**	**<0.0001**	**31.9**	**<0.001**	**10.2**	**0.041**	**32.2**	**<0.0001**
Married	50,204 (72.9)	18,653 (27.1)	61,697 (89.6)	7,159 (10.4)	48,840 (70.9)	20,016 (29.1)	60,489 (87.8)	8,367 (12.2)
Cohabiting	14,962 (68.1)	6,998 (31.9)	19,380 (88.2)	2,581 (11.8)	15,329 (69.8)	6,632 (30.2)	18,972 (86.4)	2,988 (13.6)
Religion^a^	**816.7**	**<0.0001**	**682.4**	**<0.0001**	**114.8**	**<0.0001**	**53.0**	**<0.0001**
Christianity	38,291 (68.0)	18,003 (32.0)	49,047 (87.1)	7,247 (12.9)	38,956 (69.2)	17,338 (30.8)	48,715 (86.5)	7,579 (13.5)
Islam	16,110 (78.1)	4,521 (21.9)	19,319 (93.6)	1,312 (6.4)	14,742 (71.5)	5,889 (28.5)	18,198 (88.2)	2,433 (11.8)
No religion	1,805 (74.2)	626 (25.8)	2,220 (91.4)	210 (8.6)	1,792 (73.7)	639 (26.3)	2,153 (88.6)	278 (11.4)
Other religion	1,441 (74.9)	482 (25.1)	1,683 (87.5)	241 (12.5)	1,215 (63.2)	708 (36.8)	1,719 (89.3)	205 (10.7)
Traditional/Voodoo	1,168 (78.4)	322 (21.6)	1,360 (91.3)	130 (8.7)	958 (64.3)	532 (35.7)	1,309 (87.9)	180 (12.1)
Missing (excluded from this analysis)								
Education	**754.0**	**<0.0001**	**517.4**	**<0.0001**	**227.4**	**<0.0001**	**159.7**	**<0.0001**
No formal education	22,629 (72.7)	8,486 (27.3)	28,030 (90.1)	3,085 (9.9)	21,829 (70.2)	9,286 (29.8)	27,257 (87.6)	3,858 (12.4)
Primary	19,711 (67.2)	9,621 (32.8)	25,318 (86.3)	4,014 (13.7)	20,322 (69.3)	9,010 (30.7)	25,338 (86.4)	3,994 (13.6)
Secondary	18,142 (73.2)	6,654 (26.8)	22,435 (90.5)	2,362 (9.5)	17,604 (71.0)	7,193 (29.0)	21,712 (87.6)	3,084 (12.4)
Higher	4,684 (84.0)	890 (16.0)	5,295 (95.0)	279 (5.0)	4,414 (79.2)	1,160 (20.8)	5,154 (92.5)	419 (7.5)
Occupation	**901.2**	**<0.0001**	**756.9**	**<0.0001**	**896.5**	**<0.0001**	**322.5**	**<0.0001**
Not work	20,422 (76.4)	6,322 (23.6)	24,547 (91.8)	2,197 (8.2)	20,334 (76.0)	6,410 (24.0)	23,997 (89.7)	2,747 (10.3)
Managerial	4,346 (79.7)	1,106 (20.3)	5,085 (93.3)	366 (6.7)	4,182 (76.7)	1,270 (23.3)	4,944 (90.7)	507 (9.3)
Clerical	844 (79.6)	216 (20.4)	998 (94.2)	61 (5.8)	854 (80.6)	206 (19.4)	985 (92.9)	75 (7.1)
Sales	11,154 (72.0)	4,343 (28.0)	14,067 (90.8)	1,430 (9.2)	10,399 (67.1)	5,098 (32.9)	13,358 (86.2)	2,139 (13.8)
Agriculture	17,346 (66.5)	8,732 (33.5)	22,299 (85.5)	3,779 (14.5)	17,931 (68.8)	8,146 (31.2)	22,513 (86.3)	3,565 (13.7)
Other	1,253 (72.3)	479 (27.7)	1,569 (90.6)	163 (9.4)	1,063 (61.4)	669 (38.6)	1,461 (84.4)	270 (15.6)
Household and domestic	5,075 (69.2)	2,254 (30.8)	6,461 (88.2)	868 (11.8)	4,729 (64.5)	2,600 (35.5)	6,244 (85.2)	1,085 (14.8)
Manual works	4,728 (68.3)	2,199 (31.7)	6,051 (87.4)	876 (12.6)	4,677 (67.5)	2,250 (32.5)	5,959 (86.0)	968 (14.0)
Parity	**789.8**	**<0.0001**	**147.0**	**<0.0001**	**638.1**	**<0.0001**	**390.4**	**<0.0001**
0	4,385 (81.2)	1,014 (18.8)	4,995 (92.5)	404 (7.5)	4,278 (79.2)	1,121 (20.8)	4,976 (92.1)	424 (7.9)
1	11,164 (78.0)	3,144 (22.0)	12,991 (90.8)	1,316 (9.2)	10,961 (76.6)	3,347 (23.4)	13,004 (90.9)	1,303 (9.1)
2	12,521 (72.9)	4,654 (27.1)	15,372 (89.5)	1,803 (10.5)	12,230 (71.2)	4,945 (28.8)	15,129 (88.1)	2,046 (11.9)
3	11,052 (70.4)	4,657 (29.6)	14,031 (89.3)	1,678 (10.7)	10,914 (69.5)	4,794 (30.5)	13,600 (86.6)	2,109 (13.4)
4+	26,044 (68.1)	12,182 (31.9)	33,688 (88.1)	4,538 (11.9)	25,785 (67.5)	12,441 (32.5)	32,752 (85.7)	5,474 (14.3)
Wealth index	**408.2**	**<0.0001**	**181.6**	**<0.0001**	**155.5**	**<0.0001**	**102.2**	**<0.0001**
Poorest	12,597 (69.9)	5,433 (30.1)	15,998 (88.7)	2,032 (11.3)	12,704 (70.5)	5,326 (29.5)	15,754 (87.4)	2,276 (12.6)
Poorer	12,508 (69.4)	5,505 (30.6)	15,833 (87.9)	2,179 (12.1)	12,442 (69.1)	5,570 (30.9)	15,619 (86.7)	2,394 (13.3)
Middle	12,673 (70.1)	5,414 (29.9)	16,049 (88.7)	2,038 (11.3)	12,502 (69.1)	5,585 (30.9)	15,614 (86.3)	2,473 (13.7)
Richer	13,477 (71.8)	5,285 (28.2)	16,714 (89.1)	2,048 (10.9)	13,209 (70.4)	5,554 (29.6)	16,423 (87.5)	2,339 (12.5)
Richest	13,911 (77.6)	4,013 (22.4)	16,482 (92.0)	1,442 (8.0)	13,311 (74.3)	4,613 (25.7)	16,051 (89.6)	1,873 (10.4)
Place of residence	**124.2**	**<0.0001**	**219.1**	**<0.0001**	**51.7**	**<0.0001**	**11.5**	**0.038**
Urban	25,251 (73.9)	8,918 (26.1)	31,174 (91.2)	2,996 (8.8)	24,622 (72.1)	9,548 (27.9)	30,061 (88.0)	4,109 (12.0)
Rural	39,915 (70.5)	16,732 (29.5)	49,903 (88.1)	6,744 (11.9)	39,547 (69.8)	17,100 (30.2)	49,400 (87.2)	7,247 (12.8)
Partner's age	**36.8**	**<0.0001**	**33.6**	**<0.001**	**113.0**	**<0.0001**	**80.5**	**<0.0001**
Less than 21	755 (76.3)	234 (23.7)	894 (90.5)	94 (9.5)	785 (79.4)	204 (20.6)	908 (91.8)	81 (8.2)
21–39	36,766 (71.9)	14,396 (28.1)	45,492 (88.9)	5,670 (11.1)	36,690 (71.7)	14,472 (28.3)	45,119 (88.2)	6,043 (11.8)
40–59	24,590 (71.1)	9,992 (28.9)	30,948 (89.5)	3,634 (10.5)	23,868 (69.0)	10,714 (31.0)	29,863 (86.4)	4,719 (13.6)
60 and above	3,056 (74.8)	1,029 (25.2)	3,743 (91.6)	342 (8.4)	2,826 (69.2)	1,259 (30.8)	3,572 (87.4)	513 (12.6)
Partner's education	**721.5**	**<0.0001**	**539.5**	**<0.0001**	**208.9**	**<0.0001**	**133.1**	**<0.0001**
No formal education	20,255 (74.0)	7,108 (26.0)	24,722 (90.3)	2,641 (9.7)	19,393 (70.9)	7,970 (29.1)	24,106 (88.1)	3,257 (11.9)
Primary	17,962 (67.3)	8,746 (32.7)	22,981 (86.0)	3,727 (14.0)	18,495 (69.2)	8,213 (30.8)	23,202 (86.9)	3,506 (13.1)
Secondary	19,819 (71.0)	8,095 (29.0)	25,105 (89.9)	2,809 (10.1)	19,512 (69.9)	8,402 (30.1)	24,137 (86.5)	3,777 (13.5)
Higher	7,058 (81.0)	1,653 (19.0)	8,173 (93.8)	537 (6.2)	6,699 (76.9)	2,011 (23.1)	7,911 (90.8)	800 (9.2)
Don't know	73 (60.3)	48 (39.7)	95 (78.5)	26 (21.5)	69 (57.0)	52 (43.0)	106 (87.6)	15 (12.4)

Note: Bold values indicate the *χ*² statistics and corresponding *p*-values.

High empowerment in attitudes towards violence remained associated with lower odds of IPV in both the crude and adjusted models (cOR = 0.585, 95% CI: 0.549–0.624; aOR = 0.585, 95% CI: 0.546–0.626). High social independence was also associated with lower odds in both models (cOR = 0.813, 95% CI: 0.761–0.870; aOR = 0.838, 95% CI: 0.767–0.915). Neither medium nor high decision-making empowerment was independently associated with IPV after adjustment (medium: aOR = 0.968, 95% CI: 0.895–1.046; high: aOR = 1.049, 95% CI: 0.975–1.129).

Increasing age remained significantly associated with higher odds of IPV relative to women aged 15–19 across both models, with the highest odds at 40–44 years (cOR = 2.112, 95% CI: 1.826–2.444, *p* < 0.001; aOR = 1.964, 95% CI: 1.605–2.404, *p* < 0.001). Cohabitation was significantly associated with higher odds relative to marriage in both models (cOR = 1.139, *p* < 0.001; aOR = 1.113, *p* < 0.01). Islam was significantly associated with lower odds relative to Christianity in both models (cOR = 0.859, *p* < 0.001; aOR = 0.884, *p* < 0.01).

Education showed a mixed pattern after adjustment. Compared with women with no formal education, those with primary education had slightly higher odds of IPV (aOR = 1.080, 95% CI: 1.004–1.163), while higher education was associated with lower odds (aOR = 0.751, 95% CI: 0.607–0.929). Sales (cOR = 1.399, aOR = 1.223), agricultural work (cOR = 1.384, aOR = 1.168), other work (cOR = 1.617, aOR = 1.446), household/domestic services (cOR = 1.518, aOR = 1.343), and manual work (cOR = 1.419, aOR = 1.256) were all significantly associated with higher odds of IPV relative to not working in both models (all *p* < 0.001).

Parity of 2, 3, and 4+ remained significantly associated with higher odds of IPV relative to nulliparous women in both models, with the strongest association among women with 4 or more children (cOR = 1.962, *p* < 0.001; aOR = 1.482, *p* < 0.001). The richest wealth quintile remained significantly associated with lower odds relative to the poorest in both models (cOR = 0.808, *p* < 0.001; aOR = 0.811, *p* < 0.001).

Partner's secondary education remained significantly associated with higher odds of IPV in both models (cOR = 1.158, *p* < 0.001; aOR = 1.230, *p* < 0.001). This is presented in Table 4.

**Table 4:** Survey-weighted logistic regression of factors associated with past-year IPV.

Variable	cOR (95% CI)	aOR (95% CI)
Attitude to violence		
Low empowerment	ref	ref
Medium empowerment	0.931 (0.867–0.999)*	0.907 (0.842–0.977)*
High empowerment	0.585 (0.549–0.624)***	0.585 (0.546–0.626)***
Decision-making		
Low empowerment	ref	ref
Medium empowerment	1.036 (0.961–1.116)	0.968 (0.895–1.046)
High empowerment	0.935 (0.870–1.004)	1.049 (0.975–1.129)
Social independence		
Low empowerment	ref	ref
Medium empowerment	1.002 (0.945–1.062)	0.947 (0.888–1.011)
High empowerment	0.813 (0.761–0.870)***	0.838 (0.767–0.915)***
Age		
15–19	ref	ref
20–24	1.473 (1.279–1.697)***	1.366 (1.169–1.597)***
25–29	1.745 (1.523–1.999)***	1.585 (1.340–1.876)***
30–34	1.838 (1.604–2.107)***	1.671 (1.397–1.999)***
35–39	1.839 (1.600–2.113)***	1.662 (1.367–2.020)***
40–44	2.112 (1.826–2.444)***	1.964 (1.605–2.404)***
45–49	1.883 (1.622–2.187)***	1.774 (1.437–2.190)***
Marital status		
Married	Ref	Ref
Cohabitation	1.139 (1.066–1.216)***	1.113 (1.040–1.192)**
Religion		
Christianity	Ref	Ref
Islam	0.859 (0.799–0.924)***	0.884 (0.816–0.957)**
No religion	0.829 (0.710–0.967)*	0.858 (0.732–1.006)
Other religion	0.784 (0.638–0.964)*	0.815 (0.661–1.004)
Traditional/Voodoo	0.838 (0.717–0.980)*	0.855 (0.728–1.003)
Education		
No education	Ref	ref
Primary	1.113 (1.047–1.185)**	1.080 (1.004–1.163)*
Secondary	1.003 (0.936–1.076)	1.072 (0.979–1.175)
Higher	0.575 (0.488–0.677)***	0.751 (0.607–0.929)**
Occupation		
Not working	ref	ref
Managerial	0.896 (0.787–1.020)	0.996 (0.860–1.154)
Clerical	0.664 (0.470–0.937)*	0.814 (0.553–1.200)
Sales	1.399 (1.291–1.516)***	1.223 (1.122–1.332)***
Agriculture	1.384 (1.288–1.487)***	1.168 (1.081–1.262)***
Other	1.617 (1.381–1.893)***	1.446 (1.228–1.702)***
Household/domestic services	1.518 (1.362–1.693)***	1.343 (1.201–1.503)***
Manual works	1.419 (1.287–1.564)***	1.256 (1.126–1.401)***
Parity		
0	ref	ref
1	1.177 (1.018–1.361)*	1.136 (0.972–1.327)
2	1.588 (1.377–1.830)***	1.392 (1.190–1.629)***
3	1.821 (1.585–2.092)***	1.515 (1.290–1.779)***
4+	1.962 (1.719–2.240)***	1.482 (1.253–1.751)***
Wealth index		
Poorest	ref	ref
Poorer	1.061 (0.988–1.139)	1.030 (0.956–1.109)
Middle	1.096 (1.017–1.181)*	1.025 (0.948–1.110)
Richer	0.986 (0.908–1.070)	0.912 (0.834–0.997)*
Richest	0.808 (0.736–0.886)***	0.811 (0.721–0.912)***
Place of residence		
Urban	ref	ref
Rural	1.073 (1.007–1.145)*	0.961 (0.906–1.019)
Partner's age		
Less than 20	ref	ref
21–39	1.501 (1.151–1.957)**	1.030 (0.774–1.370)
40–59	1.771 (1.356–2.312)***	1.047 (0.781–1.404)
60 and above	1.608 (1.209–2.138)**	0.915 (0.668–1.252)
Partner's education		
No education	ref	ref
Primary	1.118 (1.046–1.195)**	1.043 (0.967–1.125)
Secondary	1.158 (1.079–1.243)***	1.230 (1.130–1.338)***
Higher	0.748 (0.663–0.845)***	1.044 (0.901–1.210)
No partner/husband	1.050 (0.596–1.848)	0.948 (0.539–1.669)

cOR, crude (unadjusted) odds ratio; aOR, adjusted odds ratio; CI, confidence interval.

* *p* ≤ 0.05 (and ≥0.01).

** *p* < 0.01 (and ≥0.001).

*** *p* < 0.001.

In the country-adjusted sensitivity analysis, high empowerment in the attitudes-towards-violence and social-independence domains remained associated with lower odds of IPV, whereas decision-making empowerment remained unassociated. Some secondary associations, including those involving education, household wealth, and place of residence, were attenuated after country adjustment. Full results are presented in Supplementary Table S3.

## Discussion

This study found that the three SWPER domains did not relate to IPV in the same way. High empowerment in the attitudes-towards-violence and social-independence domains was associated with lower odds of IPV, whereas decision-making empowerment was not independently associated after adjustment. These differences reinforce the importance of treating women's empowerment as a multidimensional construct rather than relying on a single indicator.

While lower empowerment may increase a woman's vulnerability to IPV, the opposite may also be true. Women who experience IPV may become less involved in household decision-making, withdraw from social activities, or change their views about the acceptability of violence as a way of coping with ongoing abuse. As a result, lower levels of decision-making power or social independence may partly reflect the consequences of IPV rather than factors that existed before the violence occurred. Similarly, the relationship between education and IPV is difficult to interpret. Some women may have had their education interrupted by early marriage, pregnancy, or controlling partners, rather than lower educational attainment increasing their risk of IPV.

The pooled IPV prevalence of 12.4%, with country-level estimates ranging from 2.0% in Mauritania to 30.8% in Sierra Leone, highlights substantial differences across the 21 sub-Saharan African countries included in this study. This pattern is consistent with global and regional evidence showing that IPV prevalence varies widely across countries and subregions (4, 27, 28). These differences may reflect broader contextual factors, including gender norms, conflict histories, economic conditions, legal protections, institutional responses, and women's willingness or ability to disclose experiences of violence (20, 29, 30). However, the present analysis did not directly measure legal enforcement, conflict exposure, or national gender norms, so researchers should interpret these explanations as plausible contextual interpretations rather than definitive causal conclusions.

The country-adjusted sensitivity analysis broadly supported the main SWPER findings: high empowerment in the attitudes-towards-violence and social-independence domains remained associated with lower odds of IPV, while decision-making remained unassociated. This suggests that the findings were not driven solely by country composition. However, the analysis did not test country-by-SWPER interactions and therefore cannot determine whether these associations varied across countries.

The attitudes-to-violence domain emerged as the strongest empowerment-related association of IPV. Women with high empowerment in this domain had substantially lower odds of reporting IPV than those with low empowerment. This inverse association suggests that women who rejected norms used to justify wife-beating were less likely to report IPV. Women who do not perceive violence as normal, justified, or deserved may be more adept at identifying abuse, seeking assistance, and regarding controlling or violent behavior as intolerable in intimate relationships. This interpretation is consistent with evidence from low- and middle-income countries showing that acceptance of wife-beating and gender-inequitable norms are closely linked to women's experiences of IPV (8, 31). The attitudes-towards-violence domain captures whether women reject common justifications for wife-beating, but it does not directly measure control over resources, freedom from coercion, or personal safety (18, 19). Because attitudes and IPV were assessed at the same time, the observed inverse association may reflect differences in gender norms and women's agency (8, 32), but it may also be shaped by previous experiences of violence and by how women recognize or report abuse. The finding should therefore be interpreted cautiously and not taken to mean that changing attitudes alone would prevent IPV.

Unlike attitudes towards violence and social independence, decision-making empowerment showed no independent association with IPV after adjustment, with the high-empowerment estimate falling essentially at the null and marginally on the higher-odds side. A woman may be recorded as “deciding jointly” on spending or healthcare while still lacking real bargaining power, facing coercive control, or having her input overridden in practice. This means the measure can register nominal participation without capturing the underlying power dynamic that plausibly matters for IPV risk. By contrast, attitudes towards violence and social independence more directly index constructs with clearer conceptual links to IPV. Previous research likewise shows that the association between decision-making and IPV can depend on the type of decision and on whether women or men report household decision-making (33–36).

High social independence was also associated with lower odds of IPV. This domain captures women's broader social position beyond immediate household decision-making, including access to information, education, timing of marriage and childbirth, and age and education differences between partners (18, 19). The inverse association suggests that women's position in the wider social and life-course context may matter for IPV vulnerability. Women with greater social independence may have better access to information, broader support networks, and more opportunities to seek support or leave harmful relationships (37, 38). At the same time, this finding should not be interpreted to mean that social independence alone can eliminate IPV. Community norms, partner attitudes, and institutional support remain essential for women to exercise agency safely (20, 37).

Muslim women had lower adjusted odds of reporting IPV than Christian women in this study. Similar findings have been reported in multicountry research from sub-Saharan Africa and in Nigeria, where Muslim women were less likely to report IPV than Christian women (4, 39). However, evidence is not consistent across settings, as a study from Ghana found no significant difference between Muslim and Christian women (40). This variation suggests that religious affiliation is unlikely to influence IPV independently of the context in which women live. Religious affiliation is closely intertwined with country, ethnicity, culture, legislation, and socioeconomic development in this dataset, and these factors could not be fully disentangled in the present analysis. The association may reflect differences in marriage arrangements, socioeconomic conditions, gender norms, country and regional characteristics, and the willingness or ability to disclose violence. It should therefore be understood as a contextual association rather than evidence that Islam itself protects women from IPV, and should not be interpreted independently of the broader country- and ethnicity-level context in which it was observed.

Education showed a mixed pattern. Compared with women with no formal education, primary education was associated with slightly higher adjusted odds of IPV, secondary education was not associated with IPV, and higher education was associated with lower odds of IPV. The lower odds among women with higher education are consistent with evidence that additional schooling can expand women's resources and options and, in some settings, reduce later IPV (41, 42). The modest positive association at the primary level should be interpreted cautiously and does not mean that schooling increases IPV. Several explanations may account for this pattern. Women with primary education may be more likely than women with no formal education to recognize certain behaviours as abusive and to report them, so the finding may partly reflect differential reporting rather than a difference in underlying exposure. Primary education may also place women in a transitional position within changing gender norms, in which they have gained some autonomy or aspirations without a corresponding shift in their partners' attitudes or in household decision-making authority, which can heighten conflict. Changing household power dynamics as women gain even modest education may be perceived as challenging established spousal authority, particularly where broader attitudes toward gender roles remain traditional. Differential help-seeking behaviour may also play a role, as women with primary education may be more likely than those with no education to disclose violence to a survey interviewer, while remaining less likely than more highly educated women to have the resources or social support to leave a harmful relationship. Because the study pooled cross-sectional surveys conducted in different countries and years, residual confounding and unmeasured contextual differences may also partly explain the pattern (20).

Women residing in rural areas had slightly higher odds of IPV in the crude analysis. However, this association was attenuated and became non-significant after adjustment. Thus, rural residence was not independently associated with IPV in the fully adjusted model. The attenuation indicates that the crude rural–urban difference was accounted for, at least in part, by the covariates included in the model. However, because we did not perform a formal decomposition or sequential adjustment analysis, the present study cannot determine which individual covariates, or combination of covariates, contributed most to this attenuation. The finding therefore suggests that place of residence alone may be insufficient to characterize IPV risk once individual, household, and relationship characteristics are considered.

Partner's education showed a non-linear and context-dependent association with IPV. This association was attenuated to the null after adjustment. Similarly, the crude association for partner primary education was attenuated and became non-significant after adjustment. In contrast, partner secondary education remained positively associated with IPV after adjustment.

One possible interpretation is that the relationship between partner education and IPV may depend on how educational attainment translates into relationship power and social resources. Secondary education may provide partners with greater social, economic, or decision-making resources without necessarily being accompanied by changes in gender-inequitable expectations about authority within intimate relationships. Where such expectations persist, greater resources or status may not translate into more egalitarian relationships and could potentially reinforce existing power asymmetries or controlling dynamics. Previous studies suggest that the relationship between partner education and IPV may depend on the relative educational position of both partners and may vary across social contexts (43, 44).

Parity showed a graded association with IPV. Higher parity may increase caregiving and financial burden, heightening relational strain. Alternatively, women who experience IPV early may leave before further pregnancies, while those who remain continue childbearing. This could implies that higher parity could partly reflect prolonged exposure rather than a consequence of childbearing itself. The graded association between parity and IPV is consistent with studies reporting higher IPV among women with more children (45, 46). Previous research nevertheless suggests that women's ability to exercise agency safely may be influenced by relationship power, community attitudes towards violence, and access to supportive services and not just their parity (5, 20).

Taken together, the findings show why women's empowerment should be examined as a multidimensional concept rather than assumed to relate to IPV in the same way across all domains. The inverse associations for attitudes towards violence and social independence, alongside the absence of an independent association for decision-making, suggest that the domains capture different aspects of women's positions and agency. Previous research indicates that relationship power, community attitudes toward violence, and access to supportive services may influence whether women can exercise agency safely (5, 15, 20). These contextual factors were not tested as effect modifiers in the present analysis and should therefore be understood as considerations drawn from the wider literature rather than explanations established by this study.

### Strengths and limitations

This study has several strengths. It used nationally representative DHS data from 21 SSA countries, yielding a large, comparable sample of married or cohabiting women across diverse settings. It also used the SWPER Global Index, which enabled examination of women's empowerment across attitudes toward violence, social independence, and decision-making, rather than relying on a single proxy measure. The use of multivariable logistic regression strengthened the analysis by allowing adjustment for measured sociodemographic, household, partner, and residence characteristics associated with IPV. Regression diagnostics confirmed adequate model calibration, supporting the robustness of the estimates.

The study also has limitations. Its cross-sectional design means that the findings should be interpreted as associations rather than causal effects. The surveys were conducted between 2010 and 2023, and changes in legislation, conflict, education, gender norms, and IPV prevention and support services during this period may have introduced temporal heterogeneity. The pooled estimates therefore represent average associations across the included surveys rather than associations observed at a common point in time. Although the sensitivity analysis adjusted for country differences, the study did not estimate country-specific SWPER associations or test country-by-SWPER interactions. The findings should therefore not be interpreted as evidence that the associations were identical across all 21 countries.

IPV may also be underreported because of fear, stigma, social desirability bias, or limited privacy during interviews, despite the ethical and confidentiality procedures used in DHS surveys. Residual confounding remains possible because several relationship-, community-, and country-level characteristics were not available in the pooled datasets. Although DHS uses standardized questions, cultural differences in how IPV is understood and disclosed may also have affected comparisons across countries. In addition, SWPER is based on DHS items and may not capture all context-specific forms of empowerment, such as legal literacy, property ownership, access to justice, collective mobilization, or the quality of social support. Finally, country-level interpretations should be approached with caution because the study did not directly measure legal enforcement, institutional capacity, conflict exposure, or national gender norms.

### Policy implications

The findings support coordinated approaches to IPV prevention in sub-Saharan Africa. Education and access to information remain important components of women's broader social empowerment and knowledge of their rights. However, the mixed pattern observed for educational attainment in this study does not support treating education alone as a consistently protective factor against IPV. Education and rights-based information should therefore be integrated with, rather than substituted for, interventions addressing unequal power within relationships, community norms that tolerate violence, and barriers to confidential and survivor-centered support.

These efforts should be accompanied by action to address unequal power within relationships, community norms that tolerate violence, and barriers to confidential and survivor-centered support. The strong inverse association for the attitudes-towards-violence domain also indicates that norms used to justify wife-beating remain an important prevention concern (1, 8, 20, 32).

Prevention efforts should support women's ability to participate safely and meaningfully in household decisions while addressing unequal power within intimate relationships. Decision-making empowerment was not independently associated with IPV in the adjusted analysis. Nevertheless, women's participation in household decisions should be meaningful, voluntary, and free from coercion or fear of retaliation. The finding suggests that participation in selected decisions alone may not adequately represent women's power or safety within intimate relationships. Wider efforts should strengthen social support, challenge norms that tolerate violence, and improve access to confidential, survivor-centered health, legal, and psychosocial services. Governments and development partners should also enforce laws against gender-based violence and integrate IPV prevention into gender equality, education, poverty reduction, and reproductive health policies. Sustainable reductions in IPV will require coordinated action that supports women's agency while also addressing unequal relationship power, harmful social norms, and institutional barriers.

## Conclusion

In the primary pooled analysis, higher empowerment in the attitudes-towards-violence and social-independence domains was associated with lower odds of IPV, whereas decision-making empowerment was not independently associated. The country-adjusted sensitivity analysis, which included all 21 countries, broadly maintained these findings. Education showed an inconsistent pattern across the models and should therefore be interpreted cautiously. Overall, the findings show that women's empowerment is multidimensional and that its different domains do not necessarily relate to IPV in the same way. Prevention efforts should support women's agency while also addressing unequal power within relationships, norms that tolerate violence, and barriers to survivor-centered services. Because the study was cross-sectional and did not test whether the associations differed across countries, the results should be interpreted as pooled associations rather than causal or country-specific effects.

## Data Availability

Publicly available datasets were analyzed in this study. This data can be found here: https://dhsprogram.com/Data/.
