# Supplementary material for: Women's empowerment and its association with intimate partner violence in sub-Saharan Africa: a study using recent demographic and health surveys
Source: Front Glob Womens Health. 2026 Oct 7;7:1929589. doi: 10.3389/fgwh.2026.1929589 (PMC13682984; doi:10.3389/fgwh.2026.1929589)
Supplement: Supplementary file 1 [file Table1.docx]

Supplementary Table 1: Countries and the sample included in the study

| **Country** | **Sample** | **Percent (%)** |
| --- | --- | --- |
| Angola | 6,030 | 6.64 |
| Benin | 3,994 | 4.4 |
| Burkina Faso | 8,360 | 9.21 |
| Burundi | 6,389 | 7.04 |
| Cote D'voire | 3,571 | 3.93 |
| Gabon | 1,946 | 2.14 |
| Gambia | 1,648 | 1.81 |
| Ghana | 3,710 | 4.09 |
| Kenya | 10,886 | 11.99 |
| Liberia | 1,840 | 2.03 |
| Madagascar | 4,740 | 5.22 |
| Malawi | 4,500 | 4.96 |
| Mali | 3,085 | 3.4 |
| Mauritania | 2,133 | 2.35 |
| Mozambique | 2,991 | 3.29 |
| Nigeria | 8,193 | 9.02 |
| Rwanda | 1,645 | 1.81 |
| Sierra Leone | 3,700 | 4.07 |
| South Africa | 1,939 | 2.14 |
| Tanzania | 3,652 | 4.02 |
| Zambia | 5,855 | 6.45 |

Supplementary Table 2: Independent variables

| **Variable** | **Description** | **Code** |
| --- | --- | --- |
| Attitude towards violence | evaluates a woman’s view on whether a husband’s physical violence against his wife is justifiable in certain situations | 1: low empowerment, 2: medium empowerment, 3: high empowerment |
| Social Independence | examines a woman’s access to information, educational attainment, age at marriage and childbirth, as well as differences in age and education compared to her partner | 1: low empowerment, 2: medium empowerment, 3: high empowerment |
| Decision making | assesses who holds decision-making power in the household, including decisions related to the woman’s work | 1: low empowerment, 2: medium empowerment, 3: high empowerment |
| age | Age group of women | 1: 15-19, 2: 20-24, 3: 25-29, 4: 30-34, 5: 35-39, 6: 40-44, 7: 45-49 |
| marital | Marital status of women | 1: married, 2: cohabitation |
| religion | Respondent's religion | 1: Christianity, 2: Islam, 3: No religion, 4: Other religion, 5-10: unspecified |
| education | Educational level of women | 0: no education, 1: primary, 2: secondary, 3: higher |
| working | Occupational status of women | 0: not working, 1: managerial, 2: clerical, 3: sales, 4: agricultural, 5: other, 6: household, 7: manual work |
| parity | Parity of women | 0: no children, 1: one child, 2: two children, 3: three children, 4+: four or more children |
| wealth | Wealth index | 1: poorest, 2: poorer, 3: middle, 4: richer, 5: richest |
| partner age | Partner's age | 1: less than 20, 2: 21-39, 3: 40-59, 4: 60 and above |
| partner education | Partner's educational level | 0: no education, 1: primary, 2: secondary, 3: higher, 4: no partner |
| Place of residence | Whether respondent lives in a rural or urban area within their respective countries. | 1: Urban and 2: Rural |
| Country | Country of residence of respondents |  |
